# Present Conservation Status and Carcass and Meat Characteristics of Lithuanian Vištinės Goose Breed

**DOI:** 10.3390/ani12020159

**Published:** 2022-01-10

**Authors:** Violeta Razmaitė, Artūras Šiukščius, Rūta Šveistienė, Virginija Jatkauskienė

**Affiliations:** Animal Science Institute, Lithuanian University of Health Sciences, R. Žebenkos 12, LT-82317 Baisogala, Lithuania; Arturas.Siukscius@lsmuni.lt (A.Š.); Ruta.Sveistiene@lsmuni.lt (R.Š.); Virginija.Jatkauskiene@lsmuni.lt (V.J.)

**Keywords:** goose, local breed, meat, quality properties, fatty acids, lipid indices

## Abstract

**Simple Summary:**

Goose meat is known as a meat with specific aroma and flavour traits compared to other meats from poultry and other animal species. Despite a low goose meat share in meat production and consumption, a large number of different goose breeds and varieties are known. However, most of them are rare and endangered. As goose breeding is not profitable, the specificity of goose products should be evaluated and highlighted. The aim of this study was to identify the status of the conserved Lithuanian Vištinės goose breed and evaluate goose carcass and meat characteristics in the breast and thigh depending on the sex. Monitoring of geese included in the breeding system has enabled us to evaluate the changes in population size and conservation status, which remains critical maintained. The sex appeared to affect the carcass composition but did not show the effect on meat properties, whereas the muscle type had an effect on the parameters of meat and lipid quality. Thigh muscles showed more favourable lipid indices in relation to healthy nutrition of consumers in comparison to the breast.

**Abstract:**

The aim of the present study was to identify the conservation status of Lithuanian local Vištinės goose breed and evaluate the carcass and meat quality characteristics in the breast and thigh depending on the sex. The status of the Lithuanian Vištinės goose breed was evaluated by monitoring of the birds, which are included in the breeding system. Twenty geese from the nucleus flock at the age of 10 weeks and reared using a commercial concentrate diet ad libitum were used for the carcass and meat quality evaluation. Due to fluctuations in the numbers of geese and reproduction of purebred birds on a largest scale from the nucleus flock, the status of Vištinės goose population remains critical-maintained. The difference in the live body weight between males and females was insignificant, whereas males showed higher carcass weight (*p* < 0.05), including the weight of breast, wings, thighs and neck. The sex did not affect the meat quality parameters, but the anatomical location of muscles has appeared to show a high effect. Thigh muscles had lower (*p* < 0.01 and *p* < 0.001, respectively) protein and cholesterol, and higher (*p* < 0.001) intramuscular fat contents compared with the breast. Thigh muscles also showed higher (*p* < 0.001 and *p* < 0.01, respectively) pH and EZ drip loss, but lower (*p* < 0.001) cooking loss compared with the breast. The breast was characterized by a higher (*p* < 0.001) shear of force and toughness with Warner–Bratzler test and higher (*p* < 0.001) hardness detected by texture profile analysis (TPA) compared with the thigh. Breast lipids had higher (*p* < 0.001) proportion of total saturated and polyunsaturated fatty acids, whereas the thigh was characterized by a higher proportion of monounsaturated fatty acids. Atherogenic (AI) and thrombogenic (TI) indexes were higher (*p* < 0.001) and hypocholesterolemic/hypercholesterolemic (h/H) ratio was lower (*p* < 0.001) and less favourable in the breast compared with the thigh. Despite the muscle type differences, goose meat of the local conserved breed exhibited good quality and expected enhancing impact on consumer health.

## 1. Introduction

The contribution of animal production to global food and agricultural production comes from 14 animal species [1]. Besides, the main conventional, widely used farm animal poultry species, including the goose, are also important. Goose meat is a valuable food source rich in many essential nutrients. Among different products that increase the diversity of food, goose meat has a special place, being a rich and balanced gourmet ingredient delivering essential nutrients to the consumer. Apart from nutritional qualities, goose meat characterized by a special aroma and taste could provide some eating attributes that fulfill expectations not achieved by other meats [2]. Between 1970 and 2005, goose meat increased from 1.5% to 2.9% of global poultry meat production [3]. Although China was in an absolutely dominating position, as this country alone contributed 93% to global goose meat production [3,4], the largest producers of geese in Europe are Poland and Hungary [4]. Despite the fact that China is the largest producer of goose meat in the world, the volume of goose meat has recently slightly decreased [5]. High adaptability and variability of geese has resulted in a large variety of goose breeds [6]. The list of animal species and breeds used for food and agriculture includes more than 60 goose breeds from different countries [1]. However, it is known that there are significantly more different goose breeds and varieties [7,8]. The proportion of goose breeds at risk is high [9], and, therefore, in different countries efforts are being made to document goose genetic diversity and risk status [8,10,11] in order to preserve them [12,13,14,15,16].

Various birds (hens, geese, ducks and turkeys) have long been reared in Lithuania and most of them have been consumed locally. Although in 1923–1938 farmers had the largest income from the sale of geese, of which up to 854,000 head were annually exported [17], after the Second World War, during the Soviet occupation, goose farming declined and local breeds, such as Pulkinės, have become extinct and such as Vištinės have remained only in the waterfowl collection [18]. From 100 eggs of the geese from this collection, a nucleus flock of Vištinės geese was established at the Animal Science Institute in 1996 [13]. After the establishment of this nucleus flock, goslings and sometimes the eggs of Vištinės geese were consistently distributed to the other stakeholders who joined the conservation programme. Some farmers have stopped participating in the programme due to insufficient support level, but new others joined and, thus, the size of population varies greatly from year to year. The survey of the economic significance of geese in other countries reveals that small businesses on goose breeding are not typically profitable [19]. The situation in Lithuania is similar and, consequently, the consumption of goose meat is not frequent and usually limited to its consumption during Christmas, after which the size of the goose population sharply reduces. The conservation of Lithuanian farm animal genetic resources, including Vištinės geese, was based upon the primary goal to save the breeds; therefore, the sequence of activities was grounded on the formation and maintenance of the breed nucleus, investigation of breed qualities, preparation of the evaluation principles and the system and search for the possibilities of wider use [13,20]. The results have been published regarding the carcass and meat quality characteristics of different goose breeds [21,22,23,24], including some characteristics of Vištinės geese [18]; however, this was not a comprehensive assessment of meat quality.

Marketing products from local breeds are considered to be the best support of conservation programmes. Due to the fact that Lithuanian Vištinės geese are included in the programme for farm animal conservation in Lithuania, the conservation activities and efforts to increase the production from Lithuanian Vištinės geese have also caused the studies conducted on the topic. The aim of the present study was to identify the conservation status of Vištinės geese and evaluate their carcass and meat quality characteristics in the breast and tight depending on the sex.

## 2. Materials and Methods

The methods and procedures were conducted according to the National decree-law for the protection of animals used for scientific purposes, harmonized to the relevant European Union directives, and this study was approved by the review board of the Animal Science Institute of Lithuanian University of Health Sciences (protocol No. 19/01/30/).

### 2.1. Population Status of Vištinės Geese

**Figure 1:**
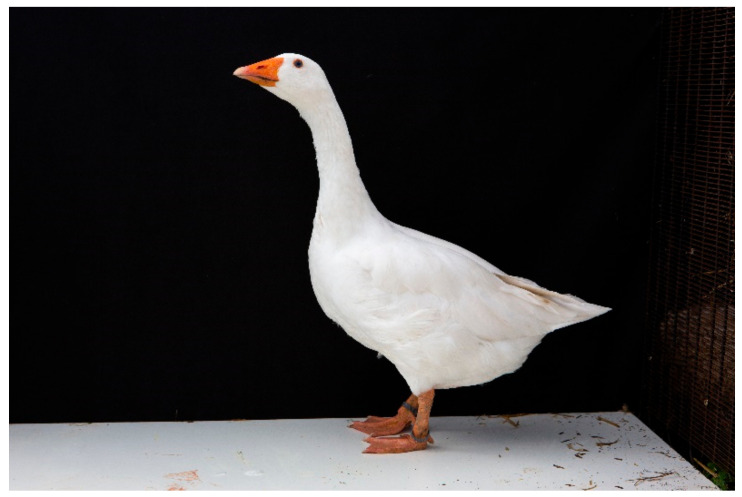
Goose of Vištinės breed. Picture made by Linas Petraška.

The status of Lithuanian Vištinės goose breed was evaluated by monitoring of the birds which are included in the breeding system. The value of critical population size is based on the current size of the active population which is kept under purebred mating scheme and the number of flocks and the region. The picture of Vištinės goose is presented in Figure 1. The effective population size (Ne) was calculated according to the formula Ne = 4 × M × F/M + F, where M is the number of males, F is the number of females.

### 2.2. Birds, Experimental Design and Slaughtering

Twenty geese of the Lithuanian native breed Vištinės at the age of 10 weeks were used for the carcass and meat quality evaluation in the study. All geese were raised under the same conditions. The unsexed goslings were kept indoors in concrete pens with sawdust bedding. After 5 weeks, the straw bedding was used. Each bird was provided with 0.38 m^2^ floor space. The birds were fed commercial concentrate diets ad libitum (starter to 3 weeks of age) and (grower from 4 weeks of age) formulated to meet the dietary requirements of geese (Table 1).

The geese selected for slaughter were weighed to determine the live body weight, transported in approximately 1 h time from the experimental farm to the accredited abattoir for waterfowl, and slaughtered 14 h after the last meal. The geese were electrically stunned followed by cutting the jugular vein and bled hanging for 5 min. Then the carcasses were scalded for approximately 1 min at approximately 63 °C, plucked, and eviscerated. The heads and legs were cut off, and the warm carcass weight, also the weight of the head, legs, heart, liver, gizzard and abdominal fat were determined. The sex of every goose (11 females and 9 males) was verified by checking the presence of testes or ovarian. The carcasses were chilled for about 24 h in a refrigerator at 4 °C, and divided into parts (neck, wings, breast, thighs and back), which were weighed.

### 2.3. Meat Quality Assessments

#### 2.3.1. Proximate Composition

The dry matter content was determined [25] by drying samples in an oven at 105 °C until a constant weight was obtained (method No. 950.46B; AOAC, 1990). The crude protein content was determined by the Kjeldahl method using the Kjeltec system 1002 apparatus (Foss-Tecator AB, Höganäs, Sweden), and a conversion factor of 6.25 was used to convert total nitrogen to crude protein (method No. 981.10; AOAC, 1990). Crude fat was determined by the Soxhlet extraction method (method No. 960.39; AOAC, 1990). Ash was determined by incineration in a muffle furnace at 550 °C for 24 h (method No. 920.153; AOAC, 1990). The content of protein, fat and ash were expressed as the weight percentage of dry matter from muscle tissues.

The cholesterol content in meat was determined according to the extraction method described by Polak et al. [26] and followed by HPLC separation and analysis on Shimadzu 10 A HPLC system (Shimadzu Corp., Kyoto, Japan). The data collection and evaluation were performed by using LC Solution (Shimadzu Corp., Kyoto, Japan) operating system. The analytical column was LiChrospher 100 RP-18e, 150 × 4.6 mm, 5 μm (Alltech Associates Inc., Columbia, MD, USA) with a guard column (LiChrospher 100 RP-18, 7.5 × 4.6 mm). The cholesterol content was expressed as mg/100 g fresh meat.

#### 2.3.2. pH Measurements

pH was measured using a digital portable pH-meter PT-380 (Boeco, Hamburg, Germany) equipped with a glass electrode (Witeg Laboratory Technik GMBH, Wertheim, Germany) after calibration using pH 4.0 and 7.0 buffer solutions. Ultimate pH (pHu) was determined 24 h after slaughter.

#### 2.3.3. Colour

The colour parameters were measured using a chromameter CR-410 Konica Minolta (Osaka, Japan) equipped with a 50 mm aperture using a C illuminant and 2° standard observer calibrated to standard white calibration plate (Y = 85.3, x = 0.3173, y = 0.3251) in the CIE L* a* b* and L* C h colour spaces (lightness, L*; redness, a*; yellowness, b*; chroma, C and hue, h) on the fresh cut surface after 30 min blooming at room temperature (18 °C).

#### 2.3.4. Water Holding Capacity

Water holding capacity was measured in two ways: drip loss and cooking loss. The drip loss was assessed according to the EZ-DripLoss method [27]. To determine the cooking loss, the frozen samples for the texture profile analysis (TPA) analysis were thawed at 4 °C for 24 h, weighed and cooked in thin-walled plastic bags at 80 °C for 1 h by immersion in a water bath with automatic temperature control [28], and then cooled at room temperature (18 ± 2 °C) and weighed again. The cooking loss (%) is defined as the difference in weight of the sample (after wiping dry) before and after cooking and cooling, divided by the sample weight at the beginning and multiplied by 100.

#### 2.3.5. Instrumental Evaluation of Texture

The texture of goose breast and thigh muscles was instrumentally measured by Warner–Bratzler shear test (WBSF) and by the texture profile analysis (TPA) using a Texture Analyser TA 1 (Measurement and Calibration Technologies Ametek Comp., Lloyd instruments, Largo, FL, USA) after cooking and cooling at room temperature (20 °C). The samples for WB test were obtained by cutting rectangles of 2 × 2 cm of cross-section, parallel to the muscle fibre direction. They were completely cut using WB shear blade with a triangular slot cutting edge and two parameters were measured: work of shear and toughness according to the following testing procedures: pre-test speed: 3 mm/s, test speed: 1 mm/min, post-test speed: 3 mm/s, triger force was 10 N. The samples for TPA were prepared and analysed by cutting rectangles of 2 × 2 cm, parallel to the muscle fibre direction and then compressing to 75%. In this test a 10 N load cell and cylindrical 20 mm-diameter probe were used. The sample was placed under the probe that moved downwards at a constant speed of 3.0 mm/s (pre-test), 1.0 mm/min (test) and 1.0 mm/s (post-test). All WB (work of shear and toughness) and TPA parameters (hardness, cohesiveness, springiness and chewiness) were measured and calculated using Lloyd Instruments Ltd. (Bognor Regis, UK) Nexygen/Ondio software together with Production Test program Version V3.0.1.

### 2.4. Fatty Acid Profiles

The extraction of lipids for the fatty acid analysis was performed with a mixture of two volumes of chloroform (Chromasolv Plus for HPLC containing 0.5–1.0% ethanol as stabilizer) and one volume of methanol as described by Folch, et al. [29]. Methylation of the samples was performed using sodium methoxide: 5 mL of 25 wt% solution in methanol was added to the sample and stirred. After 1 h, 7 mL HCL, 6 mL hexane and 2 mL H_2_O were added. The top layer was transferred into a new test-tube and evaporated. Fatty acid methyl esters were prepared according to the procedure described by Chistopherson and Glass [30]. The FAMEs were analysed using a gas liquid chromatograph (GC—2010 SHIMADZU, Kyoto, Japan) fitted with a flame ionization detector. The separation of methyl esters of fatty acids was affected on the capillary column Rt 2560 (100 m × 0.25 mm × 0.2 μm; Restek, Bellefonte, PA, USA) by temperature programming from 140 °C to 240 °C. The temperatures of the injector and detector were held, respectively, at 240 °C and 260 °C. The rate of flow of carrier gas (nitrogen) through the column was 0.79 mL/min. The peaks were identified by comparison with the retention times of the standard fatty acid methyl esters “37 Component FAME Mix” and trans FAME MIX k 110 (Supelco, Bellefonte, PA, USA). The relative proportion of each fatty acid was expressed as the relative percentage of the sum of the total fatty acids using “GC solution” software for Shimadzu gas chromatograph workstations.

### 2.5. Lipid Quality Indices

Lipid quality indices, i.e., atherogenic index (AI) and thrombogenic index (TI), were calculated according to Ulbricht and Southgate [31]. The hypocholesterolemic/hypercholesterolemic (h/H) ratio was calculated according to Fernandez et al. [32]. The peroxidizability index (PI) was determined according to Du et al. [33].

### 2.6. Statistical Analysis

The data were subjected to the analysis of variance in general linear (GLM) procedure in IBM SPSS Statistics 22 with the least significant difference tests (LSD) to determine the significance of differences of means between the groups. The GLM model included a fixed factor of goose sex and muscle anatomical location. The differences were regarded as significant when *p* < 0.05, but the differences of 0.05 ≤ *p* < 0.10 would be considered as trends.

## 3. Results and Discussion

### 3.1. Population Status of Vištinės Geese

**Figure 2:**
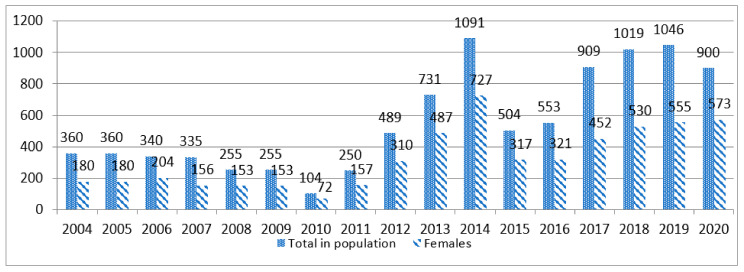
Vištinės geese numbers from 2004 to 2020.

The numbers of Vištinės geese over the last decade showed considerable changes in the population size (Figure 2). Despite the population size fluctuations, currently, in addition to the nucleus flock at the Animal Science Institute, 900 geese have already been included in the National Register of Vištinės goose breed. Beginning from 2005, farmers who keep the animals belonging to critical and endangered Lithuanian native breeds are receiving the subsidies from the Rural Development Programme, and this helped to increase the numbers in some populations [13,20]. However, the number of Vištinės geese increased only after establishing the breeding association responsible for Vištines goose herd book register in 2010 and after active dissemination of the superior genetic material from the nucleus to other farmers started. When birds are located only in one flock, there is a risk that accidents, disease outbreaks, disposal of the flock for economic, health, age or other unforeseen reasons and circumstances could increase the danger of breed disappearing.

**Figure 3:**
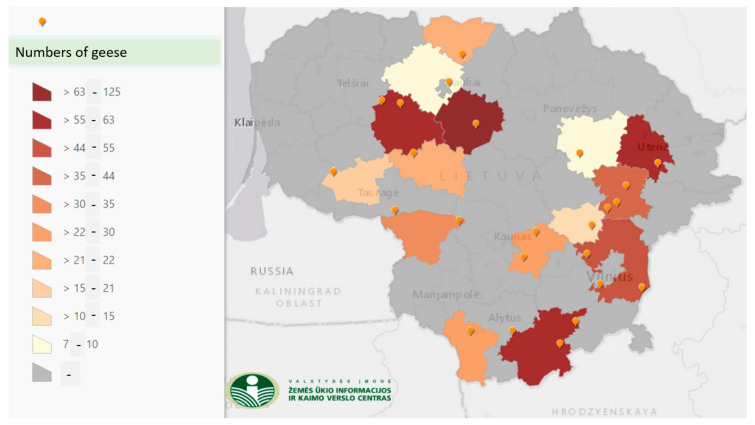
Distribution of farms rearing Vištinės goose breed and density of birds (Data from Animal breeding portal. Available in Lithuanian at https://www.vic.lt/veislininkyste/zemelapiai/ accessed on 1 September 2021).

Nowadays there are 20 associated and other farmers who keep Vištinės geese in 15 districts of Lithuania (Figure 3).

However, the reproduction of Vištinės geese on a largest scale remains in the nucleus flock. Moreover, there is lack of information about the reproduction in the breeding flocks of Vištinės geese because separate housing of 2–3 geese and an assigned gander for them has not been adopted. Therefore, it could be considered that eggs for incubation were selected using mass selection due to the lack of exact information from which birds thenew generation was derived. Moreover, there are large reductions in goose numbers after goose slaughtering for Christmas. Lack of information also prevented determination of the change in the generations during five last years as suggested by Verrier et al. [11] for poultry.

The minimum value of sex ratio is not precisely fixed but there is a provision that the sex ratio for poultry should not be below 0.2 because there is a direct association between the sex ratio and the effective population size [15]. The observed sex ratios in the conserved Vištinės goose population varied from 0.44 to 1.15 in 2007 but this did not help either to maintain stability, or to increase the population size until 2012. The minimum of effective population size (Ne = 88) was estimated in 2010. Although the maximum of Ne increased up to 970 in 2014, later the numbers of Vištinės geese and their effective population size decreased. Although the effective population size Ne is considered as the main factor in the conservation of animal genetic resources [16] and was used for status evaluation of goose breeds [7,8,15,16,34], but doubts still remain as to its full suitability for application in determining the risk status of goose breeds. According to the guidelines of FAO [35], when the desired Ne is achieved, it should not be allowed to decrease, because the Ne over a long period of time is mainly determined by the smallest effective size within that period. Therefore, according to the fluctuations in goose numbers and the fact that the number of females never exceeded 1000 and also that the effective population size during the conservation period has not been stabilized and is too small to prevent genetic loss [36], the conservation status of Vištinės goose breed can be categorized as endangered-maintained.

The activities of antropogenic factors are developing: various exhibitions are held to promote the breed and serve for education from kindergartens to university students and farmers. With the aim to reintroduce farmers in conservation activities and collaboration with breeding organizations, Lithuanian Endangered Farm Animals Breeders Association was established. As the economic efficiency of local breeds is quite low, the specificity of their products should be evaluated and highlighted.

### 3.2. Characteristics of Slaughtered Geese

Lithuanian Vištinės geese demonstrated body weight quite similar to other local goose breeds such as Polish Zatorska [21], Italian Romagnola [22] but higher than that of Czech goose [23], Chinese Yangzhou [24] and many local Turkish varieties [37,38,39] slaughtered at similar age.

Although our previous study on the sexual dimorphism of body-size measurements of Lithuanian Vištinės geese appeared to show an effect of sex on weight [40] but in the present study the difference in the live body weight between males and females was insignificant, except that males had heavier (*p* < 0.05) heads, legs and eviscerated warm carcasses (Table 2). Heavier male carcasses compared with those of females are in agreement with the findings of other authors [38,39,41]. The weights of male empty gizzard and liver were also higher (*p* < 0.01 and *p* < 0.05, respectively) compared with females. Higher weights of male liver and gizzard were also estimated in Yangzhou goose breed [24]. However, in the present study the differences in weight of variables did not show any effect of sex on their proportions in goose body.

The most valuable parts of goose carcass (breast and thighs) of both sexes accounted for less than a half-chilled carcass weight (Table 3). Although the morphometric dimensions did not show pronounced sexual dimorphism of Vištinės geese [40], the analysis of chilled carcass composition revealed that males had higher (*p* < 0.05 and *p* < 0.01, respectively) cold carcass weight, including the weight of breast, wings and thighs and neck compared with females, and this is in agreement with the results obtained for different Turkish breeds [39]. However, other authors have reported only on higher weight of male neck and wings [38].

### 3.3. Meat Quality

Neither the breast nor the thigh muscle proximate composition was affected by the goose sex (Table 4). However, some other authors have reported higher contents of moisture and protein in the breast of Yangzhou males [24] or a higher content of fat in the meat of Egyptian goose females [42]. The muscle anatomical location has appeared to show a high effect. Thigh muscles had lower (*p* < 0.01) protein and higher (*p* < 0.001) intramuscular fat contents compared with the breast. Higher fat content in the thigh of Vištinės geese agrees with a similar finding in the Polish native Rypinska breed [2].

Thigh muscles had a lower (*p* < 0.001) content of ash that shows the quantity of mineral elements and also a considerably lower (*p* < 0.001) cholesterol content than the breast. Thigh muscles having lower cholesterol content compared with the breast were also demonstrated by the local Polish Kartuska and Lubelska geese [43]. Other Polish authors [44] have found slightly lower contents (63.63–67.01 mg/100 g) of cholesterol in the breast of White Koluda geese than in the breast of Lithuanian Vištinės geese and have not reported any feeding effect on this parameter. However, local Turkish goose varieties reared on an extensive production system showed cholesterol content results being contrary to those in the present study [45]. All varieties of Turkish geese demonstrated higher values of cholesterol contents, and also, cholesterol contents in the thigh were higher (74.95–77.85 mg/100 g) than in the breast 49.95–54.7 mg/100 g).

Thigh muscles have shown higher (*p* < 0.001) pH compared with the breast (Table 5), and this is consistent with the results obtained for other different goose breeds [42,45,46].

Thigh muscles of Vištinės geese also tended to show a slightly lower (*p* = 0.066) colour lightness (L*) compared with the breast, whereas the thigh muscle of Egyptian geese showed higher lightness (L*), redness (a*) and yellowness (b*) values than the breast muscle [42]. In the present study, thigh muscles also demonstrated a higher (*p* < 0.01) EZ drip loss, but a lower (*p* < 0.001) cooking loss compared with the breast and these results are in agreement with the data obtained for Linda geese [46]. In the present study the sex showed only a low-level tendency (*p* = 0.076) to affect muscle lightness (L*) which was slightly higher in male muscles compared with females. However, other authors have determined the sex effect on geese meat pH and colour. Boz et al. [45] noted that meat pH and colour parameter values differed among goose varieties and, thus, some authors have found lower meat pH in males [24] and others in females [47] or did not determine any sex effect on geese meat pH and colour [42]. Besides, Boz et al. [45] found higher and Lewko et al. [48] lower meat lightness in males. Moreover, males also demonstrated higher redness (a*) [41,47] and higher [45] and lower [48] yellowness (b*).

The breast was characterized by a higher (*p* < 0.001) shear of force and toughness (Table 6) with Warner–Bratzler test (WBSF). The shear force results obtained for Vištinės geese in the present study are in contrast with the shear force data values found in the Huoyan goose study [49] with a higher thigh WBSF. The effect of genotype on WBSF was reported by different authors [21,50,51,52]. In the present study, the sex did not show any effect on WB parameters, and this is in agreement with the data obtained for Yangzhou geese [24,47], whereas some other authors [50,52] have reported about the obtained sex effect on WBSF.

Higher (*p* < 0.001) hardness of the breast muscle compared with thigh muscles was detected by the texture profile analysis (TPA). The other TPA parameters were also affected by the muscle anatomical location; however, the sex showed the effect (*p* < 0.05) on only goose meat cohesiveness with a higher value in females (Table 7). Most of the authors who studied goose meat have focused on WBSF test. Wołoszyn et al., [53] have found meat type effects on TPA parameters after studying the effects of various types of heat treatment, including cooking in a water bath-a method which was similarly used in the present study. However, meat type in the above study was meat with and without skin and fat, whereas in the present study goose muscles were taken from different anatomical locations. Both methods of texture analysis revealed that thigh muscles were more tender because of higher fat content and lower moisture loss during the cooking process.

### 3.4. Fatty Acid Composition

The breast lipids had a higher (*p* < 0.001) proportion of total saturated fatty acids, including individual palmitic (C16:0), stearic (C18:0) and behenic (C22:0) fatty acids, compared with the lipids in thigh muscles (Table 8). The differences in fatty acid composition between goose breeds and varieties were reported by many authors [43,45,54,55,56]. Some other breeds [43] also demonstrated higher proportions of SFA, including C16:0 in the breast, whereas others showed a higher proportion of SFA in leg muscles [57] or a similar composition of SFA in the breast and thigh [21]. The sex did not affect the composition of saturated fatty acids in the present study, and this is in agreement with the findings of the authors who evaluated fatty acid composition of local Turkish goose varieties [43]; however, adult Yangzhou females had a lower proportion of SFA and stearic fatty acid than males [58].

The Lithuanian Vištinės goose breed as well as other breeds [22,43,45,56] are characterized by a high proportion of monounsaturated (MUFA) fatty acids (Table 9).

However, the opposite results have also been reported. Linda geese reared under farm conditions exhibited a significantly lower (26,23) MUFA proportion [46]. The thighs showed higher (*p* < 0.001 and *p* < 0.01, respectively) proportions of total monounsaturated fatty acids, including individual oleic (C18:1n-9), gadoleic (C20:1n-9) and heptadecenoic (C17:1n-9) fatty acids, compared with the breast, but a lower (*p* < 0.001) proportion of vaccenic (C18:1n-7) acid, which is the only fatty acid to show a sex difference in the fatty acid composition of goose meat. Ganders demonstrated a higher (*p* < 0.05) proportion of C18:1n-7 than females. The effect of the muscle type has been found in local Polish geese with the breast having a lower proportion of MUFA than the thigh. However, Yangzou goose females [58] demonstrated higher MUFA proportions in both breast and thigh muscles.

The most abundant polyunsaturated fatty acid in goose meat was linoleic (C18:2n-6) fatty acid, followed by arachidonic (C20:4n-6) acid (Table 10). The same fatty acids were predominant in local Polish Rypińska and Garbonosa breeds [54]. Higher (*p* < 0.001) proportions of total polyunsaturated fatty acids (PUFA), including the most of individual γ-linolenic GLA (C18:3n-6), brassic (C20:2n-6), dihomo γ-linolenic DGLA (C20:3n-6), arachidonic; AA (C20:4n-6), EPA (C20:5n-3), adrenic (C22:4n-6), DPA (C22:5n-3) and DHA (C22:6n-3) acids were found in the breast than in the thigh. Only the proportion of eicosatrienoic; ETE (C20:3n-3) fatty acid was higher (*p* < 0.001) in the thigh compared with the breast. Other authors [57] have found higher proportions of PUFA in the leg muscles than in the breast.

Females exhibited a trace amount of docosadienoic (C22:2n-6) which was not found in the meat of males, however, males tended (*p* = 0.086) to show a slightly higher proportion of DPA (C22:5n-3) fatty acid than females.

Although the PUFA/SFA ratio (Table 11) was not affected either by the muscle anatomical location or the goose sex, this ratio in goose meat was above the minimum (0.4) recommended [59] and satisfied the recommendations for the consumer diet. These ratios in Lithuanian Vištinės geese were similar to those for the meat from White Kołuda strain W31 but lower than for local Polish breeds [56]. The n-6/n-3 PUFA ratio in the breast tended (*p* = 0.074) to be slightly lower than in the intramuscular fat (IMF) of the thigh. The other studies on Romagnola [22] and Linda [46] breeds exhibited higher n-6/n-3 PUFA ratios in the breast than in the thigh. The recommendations of Bellagio’s report on healthy agriculture, healthy nutrition, and healthy people indicated that the ratio (4:1) of n-6 PUFA to n-3 PUFA in the diet should be the goal [60]. It can be observed that n-6/n-3 PUFA ratios in the breast and thigh of Lithuanian Vištinės goose males and females were significantly higher than recommended. n-6/n-3 ratios in the present study were found to be higher but less favourable than in the Polish geese reared under similar conditions [56]. However, intensively reared Romagnola geese showed greater ratio (17.91) in the breast [22], whereas Linda geese demonstrated significantly higher ratios (22.18 and 28.09) in the thigh and breast [46]. The muscle anatomical location has appeared to affect the lipid quality indices. Atherogenic (AI) and thrombogenic (TI) indexes were higher (*p* < 0.001) and hypocholesterolemic/hypercholesterolemic (h/H) ratio was lower (*p* < 0.001) and less favourable in the breast compared with the thigh.

Some other authors have reported AI indexes [44,56] and similar [54] or higher TI indexes [42], but a lower h/H ratio [54]. Compared to pork [61], all lipid quality indices such as AI, TI indexes and h/H ratio of goose meat are more favourable for consumer health, however, if compared to horse meat [62], goose meat exhibits more favourable AI and h/H ratio. The peroxidizability index (PI) was higher (*p* < 0.001) of IMF in the breast.

## 4. Conclusions

The present status of Vištinės goose breed was categorized as endangered-maintained. As goose rearing has its own specific characteristics, conservation strategies and application of the effective population size calculation method require more attention of researchers.

Vištinės goose breed demonstrated high quality of carcass and meat. Males had heavier carcasses compared with females. However, the sex did not affect the quality of goose meat, but the muscle anatomical location appeared to show a high effect. Higher IMF, pH, EZ drip loss but lower cooking loss and lower toughness and hardness obtained by WBSF and TPA tests were found in thigh muscles. Intramuscular fat of thigh muscles had higher proportions of monounsaturated fatty acids and lower proportions of saturated and polyunsaturated fatty acids, as well as more favourable AI and TI indexes and h/H ratio compared with the breast. The lipid quality indices of IMF in goose meat are more favourable for consumer health than those of the most frequently used pork. Therefore, goose meat consumption should be increased and, thus, contribute to the preservation of local Vištinės goose breed. Besides the effects of genotype, muscle type and sex, the quality of goose meat and fat is also affected by rearing systems and feeds; therefore, studies on the effects of different rearing patterns should be conducted.

## Figures and Tables

**Table 1:** Nutrient composition of feed.

Components	Age of Goslings in Weeks	
	1–3	4–10
Crude protein, %	20	18
Crude fat, %	3.61	3.46
Crude fiber, %	3.69	4.05
Ash, %	5.95	6.99
Calcium, %	1.00	1.50
Phosphorus, %	0.67	0.68
Sodium, %	0.16	0.15
Lysine, %	1.00	0.90
Methionine, %	0.42	0.45
ME, kcal	2862	2800

ME-metabolizable energy.

**Table 2:** Body composition of Vištinės geese.

Variables		Sex		SED	*p*-Value
		Male *n* = 9	Female *n* = 11		
Live body weight, g		4883	4507	282.38	0.199
Eviscerated warm carcass, g		4138	3660	196.34	0.025
Dressing percentage, %		75.9	75.8	2.38	0.960
Weight and proportion in body	Head, g	174.0	149.8	8.41	0.010
	%	3.57	3.38	0.233	0.425
	Legs, g	131	111	6.99	0.013
	%	2.68	2.51	0.160	0.288
	Gut, g	227	205	15.85	0.173
	%	4.66	4.58	0.28	0.784
	Gizzard, g	215	191	14.26	0.100
	%	4.43	4.33	0.43	0.818
	Empty gizzard, g	199	167	9.94	0.004
	%	4.09	3.74	0.20	0.103
	Liver, g	77.1	67.2	4.10	0.026
	%	1.59	1.50	0.07	0.207
	Heart, g	33.0	32.1	2.57	0.730
	%	0.68	0.71	0.05	0.454
	Abdominal fat, g	81.0	76.3	13.66	0.732
	%	1.65	1.69	0.28	0.878

*p* values of GLM LSD test for sex were significantly different at *p* < 0.05 and *p* < 0.01; SED-standard error of difference.

**Table 3:** Carcass composition of Vištinės geese.

Sex	Cold Carcass Weight	Weight and Proportion in Carcass									
		Breast		Thighs		Wings		Neck		Back	
	g	g	%	g	%	g	%	g	%	g	%
Male	3442	838	25.2	717	21.6	545	16.3	299	8.9	847	25.4
Female	2996	767	24.9	658	21.5	495	16.1	254	8.3	801	26.1
SED	164.85	32.81	0.69	20.19	0.47	19.91	0.33	12.76	0.51	47.29	0.92
*p*-Value	0.014	0.046	0.774	0.009	0.931	0.021	0.512	0.002	0.198	0.354	0.429

*p* values of GLM LSD test for sex were significantly different at *p* < 0.05 and *p* < 0.01; SED-standard error of difference.

**Table 4:** Effects of muscle anatomical location and sex on proximate composition of goose meat.

Variables	Muscle		Sex		SED	*p*-Value	
	Breast (*n* = 20)	Thigh (*n* = 20)	Male (*n* = 18)	Female (*n* = 22)		Muscle	Sex
Dry matter, %	27.30	27.41	27.04	27.68	0.429	0.798	0.142
Protein, %	22.70	21.67	21.87	22.50	0.340	0.005	0.073
Fat, %	3.58	5.25	4.27	4.56	0.298	0.000	0.329
Ash, %	0.97	0.78	0.88	0.87	0.039	0.000	0.846
Cholesterol, mg/100 g	70.66	45.49	58.82	57.33	3.500	0.000	0.673

*p* values of GLM LSD test for muscle type were significantly different at *p* < 0.01, *p* < 0.001 and *p*-values of 0.05 ≤ *p* < 0.10 for sex would be considered as trend; SED-standard error of difference.

**Table 5:** Effects of muscle anatomical location and sex on meat quality parameters of Lithuanian Vištinės geese.

Variables	Muscle		Sex		SED	*p*-Value	
	Breast (*n* = 20)	Thigh (*n* = 20)	Male (*n* = 18)	Female (*n* = 22)		Muscle	Sex
pH	5.76	6.51	6.14	6.13	0.053	0.000	0.917
Colour L*	40.58	38.42	39.72	39.28	1.142	0.066	0.076
a*	16.85	16.82	16.71	16.96	0.453	0.953	0.586
b*	4.74	4.69	4.53	4.90	0.783	0.946	0.633
C	17.23	17.36	17.16	17.43	0.480	0.789	0.580
h	12.68	14.56	13.25	13.99	1.083	0.090	0.503
EZ Drip loss, %	0.84	1.34	0.98	1.20	0.182	0.010	0.244
Cooking loss, %	38.05	31.52	35.29	34.28	0.652	0.000	0.132

*p* values of GLM LSD tests for muscle type were significantly different at *p* < 0.01, *p* < 0.001 and *p*-values of 0.05 ≤ *p* < 0.10 for muscle type and sex would be considered as trends; SED-standard error of difference; lightness (L*); redness (a*); yellowness (b*).

**Table 6:** Effects of muscle anatomical location and goose sex on Warner-Bratzler test parameters.

Variables	Muscle		Sex		SED	*p*-Value	
	Breast	Thigh	Male	Female		Muscle	Sex
Shear of force, N	2.20	1.62	1.92	1.90	0.147	0.000	0.862
Toughness, N	124.80	88.87	114.48	99.18	10.664	0.001	0.157

*p* values of GLM LSD test for muscle type were significantly different at *p* < 0.001; SED-standard error of difference.

**Table 7:** Effects of muscle anatomical location and goose sex on parameters of texture profile analysis.

Variables	Muscle		Sex		SED	*p*-Value	
	Breast	Thigh	Male	Female		Muscle	Sex
Cohesiveness	2.24	2.41	2.25	2.40	0.061	0.008	0.020
Guminess, N	22.91	12.35	17.96	17.30	1.517	0.000	0.667
Hardness, N	50.95	29.44	39.51	40.88	3.399	0.000	0.689
Springiness	0.87	0.86	0.86	0.86	0.003	0.009	0.148
Chewiness, N	19.86	10.61	15.56	14.92	1.338	0.000	0.636

*p* values of GLM LSD test for muscle type and sex were significantly different at *p* < 0.05, *p* < 0.01 and *p* < 0.001; SED-standard error of difference.

**Table 8:** Effects of muscle anatomical location and sex on saturated fatty acid (% of total FA) composition of goose meat lipids.

Fatty Acids	Location		Sex		SED	*p*-Value	
	Breast	Thigh	Male	Female		Location	Sex
C12:0	0.01	0.01	0.01	0.01	0.002	0.416	0.771
C14:0	0.34	0.32	0.33	0.32	0.016	0.236	0.769
C15:0	0.03	0.03	0.03	0.03	0.002	0.096	0.878
C16:0	20.41	19.07	19.87	19.61	0.386	0.001	0.506
C17:0	0.05	0.05	0.05	0.05	0.008	0.813	0.739
C18:0	6.37	5.18	5.77	5.78	0.210	0.000	0.957
C20:0	0.05	0.04	0.05	0.04	0.004	0.066	0.230
C22:0	0.18	0.12	0.16	0.15	0.013	0.000	0.247
SFA	27.44	24.81	26.26	25.99	0.461	0.000	0.561

*p* values of GLM LSD test for muscle type were significantly different at *p* < 0.001 and *p*-values of 0.05 ≤ *p* < 0.10 would be considered as trends; SED-standard error of difference.

**Table 9:** Effects of muscle anatomical location and sex on monounsaturated fatty acid (% of total FA) composition of goose meat lipids.

Fatty Acids	Location		Sex		SED	*p*-Value	
	Breast	Thigh	Male	Female		Location	Sex
C14:1n-7	0.01	0.02	0.02	0.01	0.007	0.123	0.106
C16:1n-9t	0.02	0.03	0.03	0.03	0.003	0.206	0.635
C16:1n-9	0.27	0.24	0.26	0.25	0.015	0.083	0.687
C16:1n-7	2.76	3.24	2.96	3.04	0.122	0.000	0.511
C17:1n-9	0.11	0.18	0.14	0.14	0.024	0.005	0.845
C18:1n-9t	0.16	0.16	0.16	0.16	0.007	0.289	0.709
C18:1n-9	43.75	50.39	46.64	47.49	0.991	0.000	0.398
C18:1n-7	2.67	1.91	2.42	2.16	0.099	0.000	0.011
C20:1n-9	0.38	0.43	0.40	0.41	0.011	0.000	0.564
C22:1n-9	0.02	0.02	0.02	0.02	0.002	0.093	0.347
MUFA	50.14	56.60	53.05	53.69	0.937	0.000	0.494

*p* values of GLM LSD test for muscle type and sex were significantly different at *p* < 0.05, *p* < 0.01 and *p* < 0.001 and *p*-values of 0.05 ≤ *p* < 0.10 would be considered as trends; SED-standard error of difference.

**Table 10:** Effects of muscle anatomical location and sex on polyunsaturated fatty acid (% of total FA) composition of goose meat lipids.

Fatty Acids	Location		Sex		SED	*p*-Value	
	Breast	Thigh	Male	Female		Location	Sex
C18:2 n-6t	0.03	0.02	0.03	0.03	0.005	0.243	0.830
C18:2 n-6	13.75	13.65	13.53	13.88	0.343	0.770	0.323
C18:3 n-6	0.04	0.03	0.03	0.03	0.002	0.001	0.557
C18:3 n-3	1.01	1.01	1.01	1.01	0.034	0.835	0.836
C20:2 n-6	0.14	0.12	0.13	0.13	0.007	0.001	0.443
C20:3 n-6	0.10	0.05	0.08	0.07	0.007	0.000	0.625
C20:3 n-3	0.01	0.04	0.02	0.03	0.009	0.001	0.280
C20:4 n-6	3.65	1.70	2.86	2.49	0.283	0.000	0.200
C20:5 n-3	0.07	0.04	0.06	0.05	0.005	0.000	0.409
C22:2 n-6	0.01	0.00	0.00	0.01	0.003	0.336	0.054
C22:4 n-6	0.58	0.35	0.50	0.43	0.043	0.000	0.127
C22:5 n-3	0.24	0.13	0.20	0.17	0.016	0.000	0.086
C22:6 n-3	0.28	0.13	0.22	0.19	0.025	0.000	0.167
PUFA	19.91	17.29	18.67	18.53	0.554	0.000	0.793

*p* values of GLM LSD test for muscle type were significantly different at *p* < 0.01, *p* < 0.001 and *p*-values of 0.05 ≤ *p* < 0.10 for sex would be considered as trends; SED-standard error of difference.

**Table 11:** Total trans fatty acids and fatty acid ratios and lipid quality indexes in intramuscular fat of goose meat.

Variables	Location		Sex		SED	*p*-Value	
	Breast	Thigh	Male	Female		Location	Sex
TFA	0.22	0.21	0.21	0.21	0.010	0.362	0.992
PUFA/SFA	0.73	0.70	0.71	0.71	0.022	0.215	0.994
n-6/n-3	11.38	11.79	11.45	11.71	0.226	0.074	0.257
AI	0.27	0.24	0.26	0.25	0.007	0.000	0.392
TI	0.69	0.61	0.66	0.65	0.015	0.000	0.602
h/H	3.21	3.61	3.38	3.44	0.094	0.000	0.533
PI	38.56	27.76	34.08	32.25	1.668	0.000	0.279
UFA	2.51	1.31	2.02	1.79	0.200	0.000	0.256

*p* values of GLM LSD test for muscle type were significantly different at *p* < 0.001 and *p*-values of 0.05 ≤ *p* < 0.10 would be considered as trends; SED-standard error of difference; TFA-sum of all identified trans fatty acids; PUFA/SFA-ratio of ΣPUFA to ΣSFA, n-6/n-3-ratio of Σn-6 PUFA to Σn-3 PUFA, AI-atherogenic index, TI-thrombogenic index, h/H-hypocholesterolemic/hypercholesterolemic ratio, PI-peroxidizability index. UFA-sum of unidentified fatty acids and their isomers.

## Data Availability

Data is contained within this article.
